# Indents

**Published:** 1918-11

**Authors:** 


### INDENTS

**☞Brief Articles of Technical or Scientific Value will receive notice and comment. Contributions invited.**

---

*Pressure Anesthesia*

When I have occasion to use pressure anesthesia nowadays, I always try this experiment. Having placed my pluglet in position and moistened it, applied my soft rubber, and made the patient wince after proper preparation for what was in store, I hold the pressure at the first point, and ask the patient to note whether there is pain when I suddenly release the pressure. If there is no pain, I know from experience that the pulp is probably almost anesthetized or will be with very little more effort. If the patient reports pain on releasing my first pressure, then from experience I decide that I am dealing with an abnormal pulp. Usually an inflamed one. And these are not the cases that are the joys of dentistry. These are the pulps that you never get out whole, or as you prefer to see them come.

Take one of those obstinate pulps, or those partly calcified ones, and after trying your darndest to get it under, and having worn yourself to a frazzle in the attempt, by which time you just about know what you are up against, reach for Nature's own tooth solvent—lactic acid—pure, and with gentle touch, pass a smooth broach ever so slowly by steps down the canal, and note how wonderfully well the patient stands it compared with all the other heartrending methods at your service. Lactic acid by the way is rather viscid and you can carry it almost anywhere on a smooth broach provided you do not try to take too much of it at a time, in which case the droplets will fuse and run off.—Dr. Scheiman in August, *"Around the Table."*

*Self Reliance* The world is looking today for men who are clean, straightforward, efficient; men who know how to do their task so well that they do not have to do it over again. A man must be himself. My ideas and your ideas, and your opinions and my opinions, are worth just as much as the ideas and the opinions of another man as long as there are no kinks in them. My thinking is just as clear as any other man's thinking as long as there are no kinks in it, and my logic is just as correct as another man's logic if there are no kinks in it. I beg of you not to be imitators. Dare to be original, dare to be yourselves, dare to do the things which you find yourselves prepared to do. The man who dares to be original, who dares to be himself, who would rather be himself than offer to substitute another man's opinion with which he can not find himself in agreement, is the man who is wanted in these days. Have confidence in your ideas, once you are sure there are no kinks in your ideals, and no kinks in your thinking. As you go out, dare to be original in your work, and keep uppermost in your minds that a clean tooth never decays. If you dare to be original, cavities and crowns will enter into your lives in such a real sort of way that earning a livelihood will become a part of life itself, and great devotion to and interest in your work will be of far greater value to you as professional men than mere money-making.—Rev. Roy L. Smith, in *The Bur.*

*When to Use Pressure Anesthesia* The important contraindication which seems to have been generally overlooked is the one regarding the presence of a degree of infection in the pulp, a condition which most decidedly contraindicates its employment. It is of extreme importance in this connection to bear clearly in mind the indisputable fact that pulp exposure invariably spells pulp infection, and that unless the exposure is of very recent occurrence, the painless removal of the pulp must be acomplished by methods other than cocain pressure-anesthesia. Consequently, we argue that the only cases in which the use of cocain pressure-anesthesia is indicated are those of "fresh" exposure, and those in which, because of prosthetic reasons, a normal pulp has to be sacrificed, and will have to be bared prior to the application of the anesthetic solution.

With intra-osseous, infiltration and conductive methods of inducing local anesthesia, which are at the disposal of every practitioner (and even arsenic in selected cases), there should be no room for cocain pressure-anesthesia, except in the special cases above mentioned. The use of cocain pressure-anesthesia, without regard to the presence of infection in the pulp, must be condemned as a dangerous empiricism. The forcing of blood, contaminated by bacteria and their products, into the peri-apical structures in the endeavor to anesthetize the pulp, results in some of the peri-dental difficulties which dentists and physicians alike are endeavoring to prevent. The field for cocain pressure-anesthesia is limited and its misapplication may result in a chronic focus of infection with all its attendant complications.—Dr. Endleman, in August "*Around the Table.*"

*A New Thought About Making Plaster Models*

Making plaster models that are exact and perfect duplicates of the parts involved and at the same time strong and substantial with the thin ends and angles of the teeth reproduced absolutely perfect and of sufficient strength, and securing a model of isolated teeth standing alone at some distance from neighboring teeth; with such strength that they will not break off from the model when separating the case, or subsequently while working on the case, is often fraught with sore trials and disappointments because dental plaster does not possess desired edge and angle strength, nor sufficient strength to sustain a model of a slender tooth standing alone in many cases. The following described method will enable you to make a model possessing all the desired qualifications suggested above. Before filling the impression with plaster fill such portions of the impression in which you desire strength and accuracy, with synthetic porcelain, or Aschers Artificial Enamel or a good quality of dental cement and while it is soft and plastic insert a piece of copper wire or staple of required gauge to give strength, leaving the staples protruding out from the porcelain or enamel, or cement, whichever you use. When hardened fill up the impression with plaster so that the protruding staples will be imbedded in the plaster thus holding all parts of the model firmly together. Upon separating the case you will find the thin edges and angles of the teeth perfectly reproduced and strong, and the isolated teeth of the model firm and strong also.—H. A. Cross, in *Dental Review.*

*Use of Saccharin* Several years ago the federal government ruled that saccharin was a deleterious product, and could not be used in foods except when intended for medicinal purposes—that is, diabetic foods—in which instance its use should not exceed 0.03 gm. per day. During the recent weeks, the sugar shortage has become more and more acute and the temptation to use saccharin as a sweetening agent has consequently become greater at this time. In a ruling on the adulteration of a beverage which contained 0.01 per cent. saccharin, the Appellate Division of the Supreme Court of New York held that the use of saccharin, if fully disclosed on the label, did not constitute adulteration within the meaning of the Sanitary Code of New York. This judge, however, went further and stated that "since saccharin is not injurious to health, its use may be restricted, but can not be prohibited in the exercise of police power.''—*Journal A. M. A.*

*Distribution of Milk to Children under Three Years of Age* The city council of Paris, at the request of M. Ambroise Rendu, has decided to establish a credit of 2,400,000 francs to assure the continuance of the gratuitous distribution of milk to children who are less than three years of age.

*Aid for Shoulder Crown Technic* When adapting the gold base or fitting, upon which the wax contour is to be developed, we frequently have trouble in making a good adaptation. This difficulty may be overcome by looping a gold wire around the upper axial third of the fitting. Twist it up tightly and then drive it down to the shoulder similar to method employed for driving a hoop on a barrel. This wire loop will easily turn the trick by giving a perfect and snug fitting cap. If the wire is of 22k. or 24k. gold it may be left, *en situ*, while the crown is being developed and is finished in the usual manner.—F. W. Frahm, in *Pacific Gazette.*

*Constipation* De Castro insists that each digestion is intended to be accompanied by defecation, so that a single passage a day indicates in itself a certain amount of paresis of the bowel and some auto-intoxication. ''A glass of cold water sipped as the sun is rising utilizes the reflexes—including the peristaltic movements of the bowels—which the rising of the sun induces in the healthy organism.'' He advises setting an alarm clock to wake one in time for the sunrise, to take advantage of this moment of positive sideral influence. Habitual constipation can usually be cured in a month when the diet is restricted mainly to vegetables and fruits, with little to cause toxic action. ''Paraffin and similar preparations have only a palliative action, and it is preposterous to think of taking them all the rest of one's life."

*Securing Measure of Root for Crown* Run copper wire through the rolling mill and make a very thin ribbon and then anneal. It is readily passed around the root and beneath the gum if necessary. When in place, pinch the ends together, as is done in making orthodontic bands. It is quickly done and gives very accurate results. It can be soldered if desired.—Dr. Price, in *Oral Health.*

